# *In vitro* Selection of Probiotics for Microbiota Modulation in Normal-Weight and Severely Obese Individuals: Focus on Gas Production and Interaction With Intestinal Epithelial Cells

**DOI:** 10.3389/fmicb.2021.630572

**Published:** 2021-02-09

**Authors:** Alicja Maria Nogacka, Clara G. de los Reyes-Gavilán, Silvia Arboleya, Patricia Ruas-Madiedo, Ceferino Martínez-Faedo, Adolfo Suarez, Fang He, Gaku Harata, Akihito Endo, Nuria Salazar, Miguel Gueimonde

**Affiliations:** ^1^Department of Microbiology and Biochemistry of Dairy Products, Instituto de Productos Lácteos de Asturias, Consejo Superior de Investigaciones Científicas (IPLA-CSIC), Villaviciosa, Spain; ^2^Diet, Microbiota and Health Group, Institute of Health Research of the Principality of Asturias (ISPA), Oviedo, Spain; ^3^Functionality and Ecology of Beneficial Microorganisms, Institute of Health Research of the Principality of Asturias (ISPA), Oviedo, Spain; ^4^Endocrinology and Nutrition Service, Central University Hospital of Asturias (HUCA), Oviedo, Spain; ^5^Endocrinology, Nutrition, Diabetes and Obesity Group, Institute of Health Research of the Principality of Asturias (ISPA), Oviedo, Spain; ^6^Digestive Service, Central University Hospital of Asturias (HUCA), Oviedo, Spain; ^7^Technical Research Laboratory, Takanashi Milk Products Co., Ltd., Yokohama, Japan; ^8^Department of Food and Cosmetic Science, Tokyo University of Agriculture, Abashiri, Japan

**Keywords:** *in vitro* model, gut microbiota, probiotics, gas production, severe obesity, *Bifidobacterium*, *Lactobacillus*, SCFA

## Abstract

The intestinal microbiota plays important roles in the maintenance of health. Strategies aiming at its modulation, such as probiotics, have received a deal of attention. Several strains have been studied in different *in vitro* models; however, the correlation of results obtained with the *in vivo* data has been limited. This questions the usefulness of such *in vitro* selection models, traditionally relying on over-simplified tests, not considering the influence of the accompanying microbiota or focusing on microbiota composition without considering functional traits. Here we assess the potential of six *Bifidobacterium*, *Lactobacillus* and *Lacticaseibacillus* strains in an *in vitro* model to determine their impact on the microbiota not just in terms of composition but also of functionality. Moreover, we compared the responses obtained in two different population groups: normal-weight and severely obese subjects. Fecal cultures were conducted to evaluate the impact of the strains on specific intestinal microbial groups, on the production of short-chain fatty acids, and on two functional responses: the production of gas and the interaction with human intestinal epithelial cells. The response to the different probiotics differed between both human groups. The addition of the probiotic strains did not induce major changes on the microbiota composition, with significant increases detected almost exclusively for the species added. Higher levels of gas production were observed in cultures from normal-weight subjects than in the obese population, with some strains being able to significantly reduce gas production in the latter group. Moreover, in obese subjects all the *Bifidobacterium* strains tested and *Lacticaseibacillus rhamnosus* GG were able to modify the response of the intestinal cells, restoring values similar to those obtained with the microbiotas of normal-weight subjects. Our results underline the need for the screening and selection of probiotics in a target-population specific manner by using appropriate *in vitro* models before enrolling in clinical intervention trials.

## Introduction

Recent studies have underlined the important role of the gastro-intestinal microbiota (GIM) in the maintenance of host’s health (Thursby and Juge, 2017). Alterations on this GIM, the so-called “dysbiosis,” have been identified in several diseases (Duvallet et al., 2017). To this regard, obesity is not an exception and both compositional and functional differences between normal-weight (NW) subjects and obese patients have been repeatedly reported (Gomes et al., 2018; Vallianou et al., 2019). Although studies with severely obese individuals (OB) are scarce, accumulating evidence indicates the existence of dysbiosis in this group as well (Aron-Wisnewsky et al., 2019; Cani, 2019; Nogacka et al., 2020a). As it could be expected from the GIM differences, and very likely also due to the different dietary intakes, the fecal levels of short-chain fatty acids (SCFA) in OB subjects is also modified with regard to NW individuals (Kim et al., 2019; Nogacka et al., 2020a). These SCFA are important mediators on the GIM-host interaction, playing important roles on host’s health (Ríos-Covián et al., 2016).

The GIM represents a potential target for strategies focused on health maintenance and improvement, with the use of probiotics constituting a promising approach to this end. Probiotics are “live microorganisms that, when administered in adequate amounts, confer a health benefit on the host” (Hill et al., 2014). Several probiotic strains are being used in different functional products and a vast array of *in vitro* tests have been carried out to screen different strains. Most often, the process has been based in classical tests, such as tolerance to simulated gastrointestinal transit, adhesion to intestinal epithelial cells, co-culture with pathogens or immune cells, etc. However, in these *in vitro* tests, frequently the effect of the accompanying GIM has not been taken into account. For assessing the impact on the microbiota, several fecal culture models and simulators of the gastrointestinal tract have been used (Williams et al., 2015; von Martels et al., 2017), with compositional changes in the GIM being the main outcome in most of these studies, without considering potential functional changes. Moreover, in most cases this *in vitro* screening for potential probiotic strains was not driven by the impact on a specific target population. However, several studies have reported a high inter-individual variability in the response to probiotics, pointing out to the need for a population-specific selection (van Baarlen et al., 2011; Grześkowiak et al., 2012; Arboleya et al., 2013a,b). As a result of the use of these *in vitro* models, the correlation between *in vitro* and *in vivo* has often been limited (Vinderola et al., 2017). This lack of correlation may be partly explained by the use of over-simplified tests that do not consider the influence of the accompanying GIM, or when considering it, focusing only on its composition regardless of functional traits. The final consequence is that in spite of the huge amount of promising *in vitro* studies carried out with hundreds of microbial strains, only for a very limited number of them efficacy has been finally demonstrated in human intervention trials.

Different models of GIM-host interaction, such as co-cultivation of fecal samples or isolated GIM from different population groups or added with pro/prebiotics, with epithelial and/or immune cells, have been used (Arboleya et al., 2015; Nogacka et al., 2018, 2020a; Richards et al., 2019). Interestingly, several of these studies have demonstrated differences in the response induced by microbiotas from different population groups (Nogacka et al., 2018, 2020a) and between isolated GIM or among GIM added with probiotics or prebiotics (Arboleya et al., 2015; Nogacka et al., 2020b). These suggest that the inter-population differences on the gut microbiota may partly explain the high variability observed in the response to probiotics and the low correlation between *in vivo* and *in vitro* data.

In this work we explored the impact of different probiotic strains employing previously developed *in vitro* models, which take into account the GIM and that allow assessing the impact of the strains not just in terms of composition but also in terms of functionality on the GIM. This was achieved by monitoring, in real-time, the production of gas in fecal cultures and the interaction with HT29 intestinal epithelial cells, in addition to the study of the microbial composition and SCFA production, in two human population groups; NW and OB individuals.

## Materials and Methods

### Strains and Culture Conditions

Four *Bifidobacterium* strains (*Bifidobacterium animalis* subsp *lactis* IF20/1 [IPLA20020], *Bifidobacterium bifidum* TMC3108, *B. bifidum* TMC3115 and *Bifidobacterium longum* IF14/11 [IPLA20022] as well as two lactobacilli (*Lactobacillus gasseri* BM7/10 [IPLA20212] and *Lacticaseibacillus rhamnosus* (formerly *Lactobacillus rhamnosus*) GG [ATCC53103]) (Zheng et al., 2020) were used in this study. Frozen stocks were reactivated weekly in MRS agar (Biokar Diagnostics, Beauvois, France) supplemented with 0.25% (w/v) L-cysteine (MRSc; Sigma Chemical Co., St. Louis, MO, United States) by 48 h incubation at 37°C in an anaerobic chamber MG500 (Don Whitley Scientific, West Yorkshire, United Kingdom) under 80% (v/v) N_2_, 10% (v/v) CO_2_, and 10% H_2_ atmosphere. Two overnight passages in MRSc broth before batch culture experiments were performed. The microbial suspensions for fecal cultures were obtained by inoculating (1% v/v) fresh culture medium, incubating overnight under anaerobic conditions, centrifuging and washing twice the bacterial cells with PBS and adjusting to a final concentration of 1 × 10^10^ CFU/mL.

### Volunteers and Fecal Sample Collection

Fecal samples were obtained from nine healthy NW adults (BMI < 25 kg/m^2^) and six OB volunteers (BMI ≥ 40 kg/m^2^) recruited at the Digestive and Endocrinology and Nutrition Services, respectively, of the Asturias Central University Hospital (HUCA, Asturias, Spain). The mean age of the volunteers was 40 ± 9 and 44 ± 9 years for NW and OB subjects, respectively. All participants followed an unrestricted diet and had not taken antibiotics during the previous 6 months. The study was approved by the Bioethical Committee of CSIC and from the Regional Ethics Committee for Clinical Research of the Principality of Asturias in compliance with the Declaration of Helsinki of 1964, last revised in 2013. An informed written consent was obtained from each volunteer. Samples were collected and immediately introduced into anaerobic jars (Anaerocult A System, Merck, Darmstadt, Germany) for transportation to the laboratory within 1 h and stored at −80°C until use.

### Fecal Batch Cultures

Fecal samples were thawed at 37°C under anaerobic conditions. Then the samples were diluted 1/10 (w/v) with pre-reduced PBS and homogenized in a Lab-Blender 400 stomacher (Seward Medical, London, United Kingdom) at full-speed for 5 min and used as inocula for the fecal culture experiments. Carbohydrate-free basal medium (CFBM) (Al-Tamimi et al., 2006) was prepared and reduced overnight in anaerobic chamber one day before the batch fecal experiment. Pre-reduced CFBM was inoculated (10% v/v) with the fecal homogenate described above and then distributed into 100 mL bottles of the ANKOM RF system (ANKOM Technology, United States). The fecal cultures were allowed to stabilize overnight at 37°C in anaerobic conditions.

In brief, seven independent pH-free batch fermentations were performed for each human donor. We used as a carbon source 0.3% (w/v) of the fructooligosaccharide 1-kestose (β-Food Science Co. Ltd., Japan) which in previous experiments demonstrated to be more fermentable than other fructooligosaccharides not just by bifidobacteria and other intestinal anaerobes but also by lactobacilli (Nogacka et al., 2020b). Bacterial strains were added at a final concentration of 1 × 10^8^ CFU/mL to fecal cultures in bottles. A bottle was left without probiotic added to be used as control. The fecal cultures were then incubated under anaerobic conditions at 37°C for 24 h. Samples (1 mL) were taken in duplicate at time 0 before incubation (time 0; basal conditions) and after 24 h of incubation. These samples were centrifuged at full speed for 10 min and supernatants and pellets were stored separately at −20°C until analyses.

### pH and Gas Monitoring in Fecal Cultures

The pH of the cultures was determined with a pHmeter (SensION + PH3, HACH, Barcelona, Spain) and was considered as an indicator of the progression of fermentation. The cumulative gas produced along the different fermentations was monitored in real-time by using the ANKOM RF system. This system provides the increases in pressure (psi) which can be converted to mL of gas produced using the Ideal Gas Equation:

(1) V=Vj⋅Ppsi⋅⁢0.068004084

where: V = gas volume at 39°C in mL, Vj = headspace of digestion jar (Glass Bottle) in mL, Ppsi = cumulative pressure recorded by Gas Monitor System software.

The data of gas production were fitted to modified-Gompertz equation, a model frequently used to fit data of bacterial, plant growth, tumor proliferation and gas production (Ware and Power, 2017), by using the formula:

(2) $$y=A\,\times \exp\left\{-\exp\left[\frac{\mu \times \,e}{A}\,\left(\lambda -t\right)+1\right]\right\}$$

In which variables: “A” represents the upper asymptote (mL), “μ” is the rate of gas production (mL/h) and “λ” is the time lag before exponential phase (h).

### Microbiota Composition and SCFA Quantification

DNA was extracted from the bacterial pellets by using the QIAamp DNA Stool Mini kit (Qiagen GmbH, Hilden, Germany) as previously described (Nogacka et al., 2020a) and the isolated DNA was stored at −20°C until use. The absolute levels of relevant intestinal microbial groups (*Bacteroides*-*Prevotella*-*Porphyromonas* group, *Lactobacillus*-group, *Akkermansia*, *Clostridium* cluster XIVa, *Bifidobacterium* and *Faecalibacterium* genera) as well as total bacteria were determined at 0 and 24 h of fermentation by qPCR using previously described primers and conditions (Valdés et al., 2017). Variations in the levels of the species *B. longum, B. bifidum*, *B. animalis*, *Bifidobacterium adolescentis* and *Bifidobacterium catenulatum* were assessed as described elsewhere (Salazar et al., 2015; Arboleya et al., 2020). In order to investigate microbial changes as regards to the basal microbiota, the data were expressed as the log-ratio of the Fold Change before (time 0) and after 24 h incubation with different probiotic strains.

The analysis of SCFA was performed by Gas Chromatography (GC) in the fecal culture supernatants (CS) in order to determine the molar concentrations of three main compounds: acetic, propionic and butyric acids. The remaining SCFA, namely iso-butyric and iso-valeric acids were also quantified and summed up (BCFA) for further analysis. Briefly, 0.25 mL of the culture supernatants were mixed with 0.3 mL methanol, 0.05 mL of an internal standard solution (2-ethylbutyric 1.05 mg/mL), and 0.05 mL of 20% formic acid. This mixture was centrifuged and the supernatant was used for quantification of SCFA by GC as described previously (Nogacka et al., 2018). Samples were analyzed in triplicate. Increments in molar concentration of SCFA with respect to the time 0 were calculated for each fermentation batch with the different probiotic strains tested.

### Monitoring the Interaction of Intestinal Microbiotas and Fecal Culture Supernatants Supplemented With the Probiotics With HT29 Cells

We also aimed at evaluating the impact of the addition of probiotic strains on the interaction between the gut microbiota and enterocytes. To this end, we purified the microbiotas of the volunteers as described by Nogacka and co-workers (2018) and added them with the probiotic strains to be tested. In order to prevent acidification, which could damage the HT29 cells, these microbial mixtures were inactivated by UV light (Nogacka et al., 2018) and the interaction with confluent HT29 cells monolayers was evaluated by using a real-time cell analyser (RTCA-DP) xCelligence apparatus (ACEA Bioscience Inc., San Diego, CA, United States). Variations in HT29 cell monolayer trans-epithelial resistance (due to changes in morphology and/or attachment of the epithelial cells) during exposure to the microbiotas and CS were assessed. The culture conditions and the maintenance of the intestinal epithelial cell line HT29 (ECACC 91072201) is detailed in a previous work where the functional model was developed (Nogacka et al., 2018). For this functional assessment, each strain was mixed with the isolated microbiota in a bacterial proportion 1:1. Then, a ratio 10:1 of the total bacteria (6.5 × 10^7^ cells/mL) with respect to the epithelial cell was added in McCoy’s medium.

The functional assessment of the fecal CS on the behavior of HT29 cells monolayers was assessed by using filtered CS collected after 24 h of the fecal culture (pH adjusted to 7.55 ± 0.05) and diluted at 40% with McCoy’s medium. Additionally, several controls consisting on basal microbiota, and McCoy’s medium without bacteria or fecal supernatants added, were included in each experiment. Each sample was tested by duplicate using two independent E-plates. The monitoring was followed for every 10 min under standard incubation conditions. CI values recorded were normalized by the time of the sample addition and by the control sample, as previously described (Valdés et al., 2015). For statistical comparison purposes the “Area Under the Curve” (AUC), representing the CI values along 10 h of incubation for each sample, was calculated as explained in Nogacka et al. (2018).

### Statistical Analyses

Unless otherwise specified, all experimental data are reported as mean ± standard deviation. Statistical analysis of results was performed using the software SPSS v.25 (SPSS Inc., Chicago, United States). Data were compared for the effect caused on the parameters analyzed by the addition of different probiotic strains in fecal cultures from each population cohort (NW and OB) at the end of fermentation (24 h). For variables with a normal distribution (Shapiro-Wilk test) and homoscedasticity (Levene test), one way ANOVA followed by post-hoc DMS comparison were conducted. In the remaining cases (variables showing non-normal distribution), a Kruskal-Wallis test followed by a post-hoc Dunn’s test of pairwise comparisons were applied when necessary. A significant *p*-value of 0.05 was used for the interpretation of results. For two-group comparisons between OB and NW subjects, a two-tailed Student’s *t*-test or Mann-Whitney’s *U* test was conducted for the evaluation of data by parametric or non-parametric contrast, respectively.

## Results

### Effect of the Probiotic Strains on pH and Gas Production in Fecal Cultures

Drops of pH observed in fecal cultures were similar between the two human populations studied, OB and NW subjects, with values ranging from 1.42 to 1.78 pH units (Table 1). However, the response to the different probiotics showed noticeable differences between both groups. Whilst in the OB group no statistically differences were observed among treatments (control or the different probiotic strains), in NW subjects the strains *B. bifidum* TMC3115, *B. longum* IF14/11 and *L. gasseri* BM7/10 induced significantly (*p*-value <0.05) higher decreases of pH than those found in the control culture. The opposite trend was observed for gas production; thus, whilst in OB subjects some strains were able to significantly (*p*-value <0.05) reduce gas production in comparison to the control, no significant differences among treatments were detected in fecal cultures of the NW group (Table 1). Interestingly, in all the conditions tested (control and the different probiotics) fecal cultures of NW subjects shower higher gas production ability than cultures of OB individuals, although no statistical significant differences were found between cultures of both groups. In addition, the inter-individual variability was higher in NW than in the OB group, the later subjects resulting quite homogeneous in terms of gas production in fecal cultures. The kinetic parameters analyzed confirm this observation, with lower production rates in OB than in NW subjects (Table 1).

**TABLE 1:** Cumulative gas produced (mL) and decreases of pH values (Δ pH) after 24 h of incubation in fecal cultures from normal-weight (NW) and severely obesity (OB) individuals.

Group	Probiotic	Δ pH	Cumulative gas	*A*	μ	*R*^2^
OB	Control	−1.54 ± 0.18	19.47^b^ ± 4.68	19.862	1.587	0.990
	*B. animalis* IF20/1	−1.57 ± 0.24	12.97^a^ ± 2.16	15.102	1.078	0.997
	*B. bifidum* TMC3108	−1.67 ± 0.11	14.22^ab^ ± 0.82	14.004	1.181	0.996
	*B. bifidum* TMC3115	−1.71 ± 0.17	13.69^a^ ± 2.96	13.555	1.114	0.997
	*B. longum* IF14/11	−1.78 ± 0.09	12.87^a^ ± 1.62	12.637	1.127	0.997
	*L. gasseri* BM7/10	−1.74 ± 0.17	14.40^ab^ ± 1.63	14.215	1.286	0.995
	*L. rhamnosus* GG	−1.70 ± 0.17	15.54^ab^ ± 2.80	15.441	1.335	0.996
NW	Control	−1.42^a^ ± 0.18	24.37 ± 12.28	24.623	2.228	0.999
	*B. animalis* IF20/1	−1.49^ab^ ± 0.18	22.01 ± 9.84	21.918	2.192	0.999
	*B. bifidum* TMC3108	−1.63^*abc*^ ± 0.07	20.69 ± 9.11	20.154	2.470	0.997
	*B. bifidum* TMC3115	−1.64^c^ ± 0.07	19.49 ± 7.20	19.108	2.248	0.998
	*B. longum* IF14/11	−1.64^bc^ ± 0.18	18.88 ± 8.46	18.629	2.121	0.998
	*L. gasseri* BM7/10	−1.63^bc^ ± 0.13	16.67 ± 6.75	16.431	1.783	0.996
	*L. rhamnosus* GG	−1.58^*abc*^ ± 0.16	22.35 ± 11.07	22.371	2.168	0.998

*Kinetic parameters were determined using the modified-Gompertz equation, in which “A” represents the upper asymptote (mL) and “μ” is the rate of gas production (mL/h). The values not sharing the same superscript (a, b, or c) indicate significant differences (*p*-value <0.05) among probiotic strains and/or control for fecal cultures from each population group (NW or OB).*

### Effect of the Probiotic Strains Addition to Fecal Cultures on the Intestinal Microbiota

**FIGURE 1:**
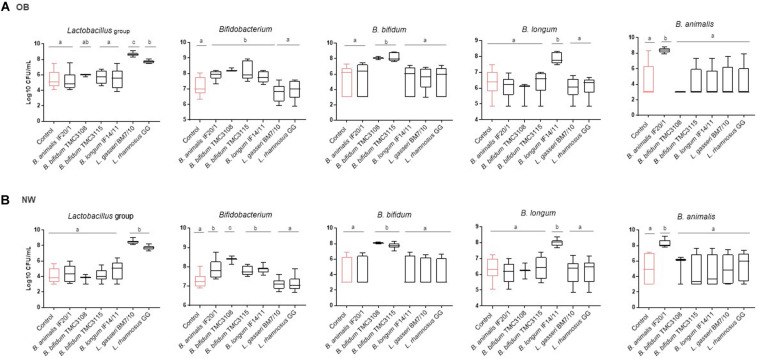
Absolute levels (Log_10_ CFU/mL) of intestinal microbial groups determined by qPCR in fecal cultures of **(A)** OB and **(B)** NW subjects. For each condition, the box and whiskers plot represent median, interquartile range and minimum and maximum values obtained in each human group (NW or OB). Different letters above the boxes indicate significant differences (*p*-value <0.05) among probiotic strains for the microbial groups considered.

The probiotic strains tested induced changes in the microbiota of fecal cultures, mostly linked to increases in the administered bacterial group or species (Figure 1 and Supplementary Table 1). As it could be expected, the addition of *Lactobacillus*/*Lacticaseibacillus* strains increased the levels of lactobacilli in the fecal culture, whereas the administration of *Bifidobacterium* strains increased bifidobacteria, and specifically the species used, regardless of the human population group. Regarding the other intestinal bacterial groups assessed, no effect of the addition of the probiotics, neither in cultures of OB nor in those of NW subjects, was observed for the levels of *Akkermansia*, Enterobacteriaceae, *Bacteroides*-group, *Faecalibacterium* or total bacteria (Supplementary Table 1). As with regard to *Clostridium* cluster XIVa the levels were not affected by the probiotic strains in fecal cultures from OB subjects, but in the NW group *B. bifidum* TMC3115 and *L. gasseri* BM7/10 promoted lower levels of this bacterial group when compared to the control culture (Supplementary Table 1).

Considering the basal differences existing on the microbial composition between fecal samples of OB and NW subjects, the fold change in bacterial levels were calculated for the different probiotics in fecal cultures of both groups of individuals. Interestingly, when the response to the different probiotic strains in the cultures of the two human groups studied were compared some statistically significant differences were found (Supplementary Figure 1). *B. animalis* subsp *lactis* IF20/1 and *B. bifidum* TMC3115 induced significantly higher increments (*p*-value <0.05) in the levels of *Bifidobacterium* in cultures of OB than in those of NW subjects. A similar trend, although not reaching statistical significance (*p*-value <0.1), was observed for *B. longum* IF14/11. Whereas *B. bifidum* TMC3108 also showed a trend (*p*-value <0.1) toward a larger reduction on the levels of *Clostridium* cluster XIVa in the OB than in NW group. Regarding the *Lactobacillus*/*Lacticaseibacillus* strains tested, *L. rhamnosus* GG led to an increase on lactobacilli levels which resulted significantly higher (*p*-value <0.05) in cultures of NW than in cultures of OB, and the same trend (*p*-value <0.1) was also observed with *L. gasseri* BM7/10. These results indicate that the increases in the corresponding groups induced by the probiotic *Lactobacillus*/ *Lacticaseibacillus* and *Bifidobacterium* strains tested, is not limited to the increase of the administered strain. The difference in the increases induced by the same probiotic observed between OB and NW groups suggest that the probiotics may also affect the intestinal populations of lactobacilli or bifidobacteria in a way that depends on the basal microbiota, known to be different for OB and NW subjects.

**FIGURE 2:**
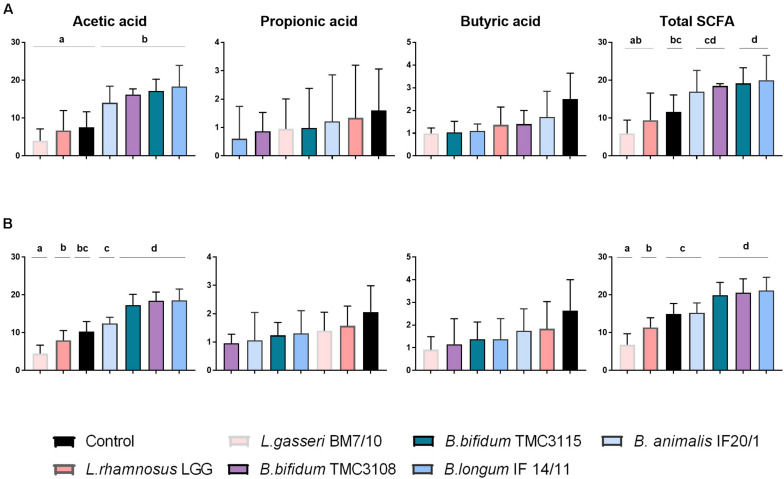
Increments in ascending order, with respect to time 0, in the concentration (mM) of the major short-chain fatty acids (acetic, propionic, and butyric acids) after 24 h of incubation with different probiotic strains in fecal cultures from OB **(A)** and NW **(B)** groups. Differences are shown for each SCFA, columns that do not share the same letter are significantly different (*p* < 0.05).

When we assessed the production of SCFA no major differences were observed in the response to the different probiotics between OB and NW fecal cultures. In cultures of both groups of individuals, acetic acid was the SCFA present at higher concentration, with propionic and butyric acids being detected at lower levels (Figure 2). No differences among probiotic strains or between these ones with respect to the control culture were observed for propionic and butyric acid. However, differences became apparent for acetic acid, with the four *Bifidobacterium* strains tested (*B. animalis* subsp *lactis* IF20/1, *B. bifidum* TMC3108, *B. bifidum* TMC3115 and *B. longum* IF14/11) inducing the production of larger amounts of acetic acid (*p*-value <0.05) than the lactobacilli, or the control culture, for OB individuals. Moreover, these four strains led to the production of a higher concentration of acetic acid (*p*-value <0.05) than the lactobacilli also in NW subjects, with the increments from *B. bifidum* TMC3108, *B. bifidum* TMC3115 and *B. longum* IF14/11 being significantly higher (*p*-value <0.05) than those obtained for all the other experimental conditions in this NW group. Given that acetic acid is clearly the predominant SCFA in the samples, these observations are mirrored as well when the total level of SCFA was considered (Figure 2).

### Effect of the Fecal Culture Supernatants and Microbiotas Added With Probiotic Strains on the Interaction With HT29 Cells

**FIGURE 3:**
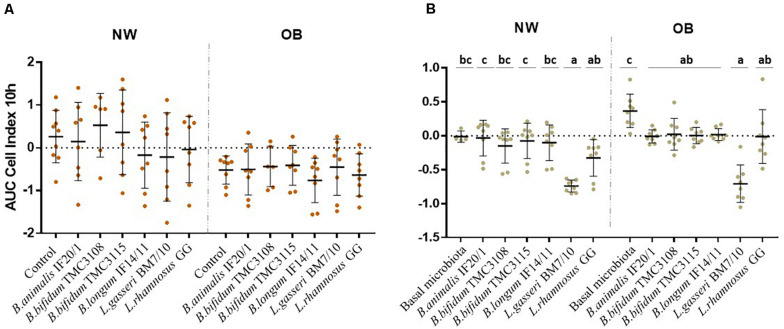
Real-time monitoring the interaction with HT29 intestinal epithelial cells between **(A)** supernatants obtained after fecal culture with probiotic and **(B)** a mixture of a probiotic strain with the gut microbiota from NW and OB population groups. Values (media ± SD) correspond to the AUC resulting from monitoring Cell Index (CI) during 10 h. Significant differences (*p*-value <0.05) represent the comparison of results before and after probiotic addition in each condition.

The interaction with intestinal cells monolayers was monitored in real-time using the RTCA system as a proxy for determining the impact of the probiotics on the functional response of the intestinal epithelium to the microbiota. This system allows monitoring in real time the epithelial cell monolayer structure/integrity by measuring the impedance and detecting changes in this parameter that may be due to changes in the morphology of the cells or in their attachment. We assessed first the effect of CS obtained at 24 h of incubation. The response observed with the supernatants of OB cultures displayed lower AUC values than those with cultures obtained from NW donors, which could be reflecting the significant difference found (*p*-value <0.05) between the control conditions from both human groups (Figure 3A). However, within cultures from each human group no statistically significant differences were observed among the different probiotics, with a similar response against all the strains tested (Figure 3A). Next, we assessed the effect of the addition of the different strains to the basal microbiota of the OB or NW individuals (Figure 3B). Again, differences (*p*-value <0.05) were observed between the basal microbiota of OB and NW subjects, with the microbiotas from OB subjects showing higher AUC values than that of NW individuals. When the different strains were compared, the *Lactobacillus*/ *Lacticaseibacillus* strains displayed lower AUC values than the bifidobacteria in the NW group, whereas the results reached statistical significance (*p*-value <0.05) only for *L. gasseri* BM7/10 in NW subjects. Moreover, in OB subjects all bifidobacteria and *L. rhamnosus* GG strains induced significantly lower (*p*-value <0.05) AUC values than that obtained for the basal microbiota without probiotics added (Figure 3B), reaching values similar to those obtained for the microbiota of NW subjects.

## Discussion

Restoration of the eubiotic condition of the GIM is of great interest for the prevention of different diseases and probiotics have been among the most used tools to this end (Sanders et al., 2019). However, in spite of the several beneficial effects attributed to probiotics only some of them have been substantiated by clinical evidence (Merenstein et al., 2020). Therefore, in Europe with the sole exception of the yogurt and lactose intolerance, no other probiotic-based health claim has been approved. This is in contrast to the high amount of *in vitro* studies claiming the probiotic potential of several strains (Vinderola et al., 2017). This lack of agreement suggests that the conventional and perhaps over-simplified *in vitro* models most used until now in probiotics research (adhesion to mucus or intestinal cells, tolerance to acid and bile, pathogen inhibition or immune modulation etc.) show a poor predictive value for the *in vivo* situation where other microorganisms are present, at very large amounts, in the ecosystem. Actually, most often the strains have been tested alone, without the accompanying microbiota which in addition may be differ among population groups. This may explain why in some cases the experiments may have failed in the identification of the most suited strains for a given target population. The use of fecal culture models, or intestinal simulators, has been also common in the search for prebiotics with microbiota modulating abilities (Roberfroid et al., 2010; Shen et al., 2011; Bajury et al., 2018; Nogacka et al., 2020b). However, such models have not been so extensively used in probiotics research. Fecal culture models, similar to those applied in this study, have been used for the selection of potentially probiotic strains for GIM modulation in newborns (Arboleya et al., 2013b) or elderly (Valdés et al., 2017), among others. However, in contrast with the current work, in most of the available studies the selection was based exclusively on the effects upon the GIM composition, without taking into consideration the functionality of the microbiota. Only a few *in vitro* anaerobic bacterial co-culture systems that consider host-gut microbiota interaction have been employed for that purpose (von Martels et al., 2017), moving from the simplest ones, as the Host-Microbiota Interaction (HMI) and the Human oxygen Bacteria anaerobic (HoxBan) models, to the most sophisticated ones such as the microdevices Gut-on-a-chip and the Human Microbial crosstalk model (HuMiX). In the present work, affordable and simple *in vitro* tests for the screening and selection of probiotics in a target-population specific manner were assessed. We monitored, in real-time, the production of gas in fecal cultures and the interaction with intestinal epithelial cells of the culture supernatants or the isolated microbiotas from NW and OB individuals supplemented with six *Bifidobacterium*, *Lactobacillus* and *Lacticaseibacillus* strains. Our *in vitro* models have taken into consideration not only the composition but also the functionality of the basal gut microbiota from NW and OB volunteers demonstrating their different response after probiotic administration.

Higher levels of gas production were generally achieved in fecal cultures from NW subjects than in the severely obese population, which is in good agreement with previous observations (Nogacka et al., 2020b). This result underlines the existence of functional differences between the GIM of NW and OB subjects and suggests a metabolically less active microbiota in OB subjects. Changes in the gas production are related with differences in the composition and metabolic activity of the basal intestinal microbiota. Additionally, prebiotics are known to affect microorganisms of the intestinal microbiota such as bifidobacteria and lactobacilli that produce acetate and lactate; these compounds could be involved in cross-feeding mechanisms with gas-producing microorganisms such as *Clostridium* and sulfate-reducing bacteria (Sarbini and Rastall, 2011). We screened different probiotic strains for their ability to influence *in vitro* the GIM composition and activity. To this end, we evaluated gas production over the fecal culture in real-time using the ANKOM RF technology. Although this method had been already applied to fecal cultures (Rotbart et al., 2018; Yao et al., 2018; Nogacka et al., 2020b), this is the first time that it is applied in the evaluation of probiotics. This real-time monitoring of gas production has made possible to discriminate between probiotics according to their different ability to modulate gas production in fecal cultures of severely OB subjects, with *B. bifidum* TMC3115, *B. animalis* IF20/1 and *B. longum* IF14/11 being able to reduce the production of gas as compared with the control culture with no probiotics added. The fecal cultures of the NW individuals presented greater heterogeneity than those of OB individuals, which may partly explain why we failed to obtain statistically significant differences in the NW population.

As with regard to the response of the GIM to the addition of the different strains tested in fecal cultures, in general we did not observe any major changes in the absolute levels of the microbial groups analyzed, with significant increases only detected for the species of the added probiotics. The sole exception was the microbial group *Clostridium* XIVa in NW individuals with the strain *B. bifidum* TMC3108, which promoted higher levels of this microbial group than the other strains. In contrast, *B. bifidum* TMC3115 and *L. gasseri* BM7/10 were able to reduce the levels of this microbial group when compared to the control cultures from NW subjects. Interestingly, the *Lactobacillus*/ *Lacticaseibacillus* strains tested led to higher levels of lactobacilli in cultures from NW than in those from OB subjects, whereas the contrary occurred for bifidobacteria. These differences are likely due to the distinct basal microbiota between groups. Indeed, when comparing lactobacilli and bifidobacteria levels between both groups of individuals, at time zero, we observed that the fecal cultures from OB subjects showed significantly higher levels of lactobacilli and lower of bifidobacteria (5.14 ± 1.07 and 6.44 ± 0.37 Log_10_ CFU/mL, respectively) than those from NW individuals (3.9 ± 0.75 and 6.91 ± 0.41 Log_10_ CFU/mL, respectively). Moreover, these observations suggest that, regardless of the strain, the genus to which the probiotic strain belongs may constitute a first choice for selecting the best probiotic for microbiota modulation in a certain target population, i.e., OB or NW. These different responses observed between fecal cultures from both population groups underline previous studies reporting that the composition of the basal microbiota conditions the response to the probiotic (Arboleya et al., 2013a; Maldonado-Gómez et al., 2016; Hou et al., 2020).

Regarding SCFA, we did not observe mayor differences in the response to the different probiotics between fecal cultures of both human groups. As expected in both cases, NW and OB, acetic acid was the main SCFA followed by propionic and butyric acids. All the *Bifidobacterium* strains tested led to greater increases in the total SCFA, and of acetic acid, than the strains of lactobacilli, with *B. bifidum* TMC3108, *B. bifidum* TMC3115 and *B. longum* IF14/11 being those promoting higher values.

Finally, we studied the interaction with intestinal epithelial cells of the CS or the isolated microbiotas from NW and OB individuals supplemented with probiotics. No differences among the probiotics tested was observed when the CS were assessed, whereas GIM added with the different strains showed clear differences among them. Lactobacilli showed lower AUC values than bifidobacteria. Interestingly in OB but not in NW subjects all tested strains were able to down-regulate the HT29 cells response to the basal microbiota of these subjects. This is interesting given that the response to the basal microbiota of OB individuals was higher when compared with that of NW subjects. Therefore, the addition of the probiotic strains was able to restore this elevated response observed in the OB group bringing this functional response back to the levels observed in NW subjects.

Our results from *in vitro* models, although performed with a low number of samples, underline the need for the study and selection of probiotics in a target-population specific manner. The effects of strains in the fecal microbiota of NW individuals may be different from those that occur in OB, as it is the case of the data reported here. This complexity is often not considered in models where the strains are studied in isolation, without taking into account the mediation of the surrounding microbiota in the final effect on the host. It is, thus, necessary to select microorganisms with large functional capacity, as a previous step to carrying out human studies that entail a high economic cost. To achieve this, affordable *in vitro* study models such as those used here are necessary allowing the identification of the strains of potential interest, such as *B. bifidum* TMC3115, for its application in a specific human population group such as severely obese subjects. Nevertheless, it has to be taken into consideration that any *in vitro* screening for potential probiotic strains will require of later human clinical trials with higher number of individuals to evidence efficacy.

## Data Availability Statement

The original contributions presented in the study are included in the article/Supplementary Material, further inquiries can be directed to the corresponding author/s.

## Ethics Statement

The studies involving human participants were reviewed and approved by the Bioethical Committee of CSIC and from the Regional Ethics Committee for Clinical Research of the Principality of Asturias. The patients/participants provided their written informed consent to participate in this study.

## Author Contributions

CR-G, FH, GH, AE, NS, and MG conceived and designed the study. AS and CM-F recruited the volunteers and obtained the samples. AN and PR-M conducted the research. AN, CR-G, SA, NS, and MG analyzed the data. MG wrote and prepared the original draft. AN, CR-G, and NS helped with the manuscript review. All authors have read and approved the final version of the manuscript.

## Conflict of Interest

FH and GH were employed by the company Takanashi Milk Products Co., Ltd. The remaining authors declare that the research was conducted in the absence of any commercial or financial relationships that could be constructed as potential conflict of interest. The authors declare that this study received funding from Takanashi Milk products. The funder had the following involvement with the study: study design.
