# Supplementary figures and images for: In vitro Selection of Probiotics for Microbiota Modulation in Normal-Weight and Severely Obese Individuals: Focus on Gas Production and Interaction With Intestinal Epithelial Cells
Source: Front Microbiol. 2021 Feb 9;12:630572. doi: 10.3389/fmicb.2021.630572 (PMC7899977; doi:10.3389/fmicb.2021.630572)

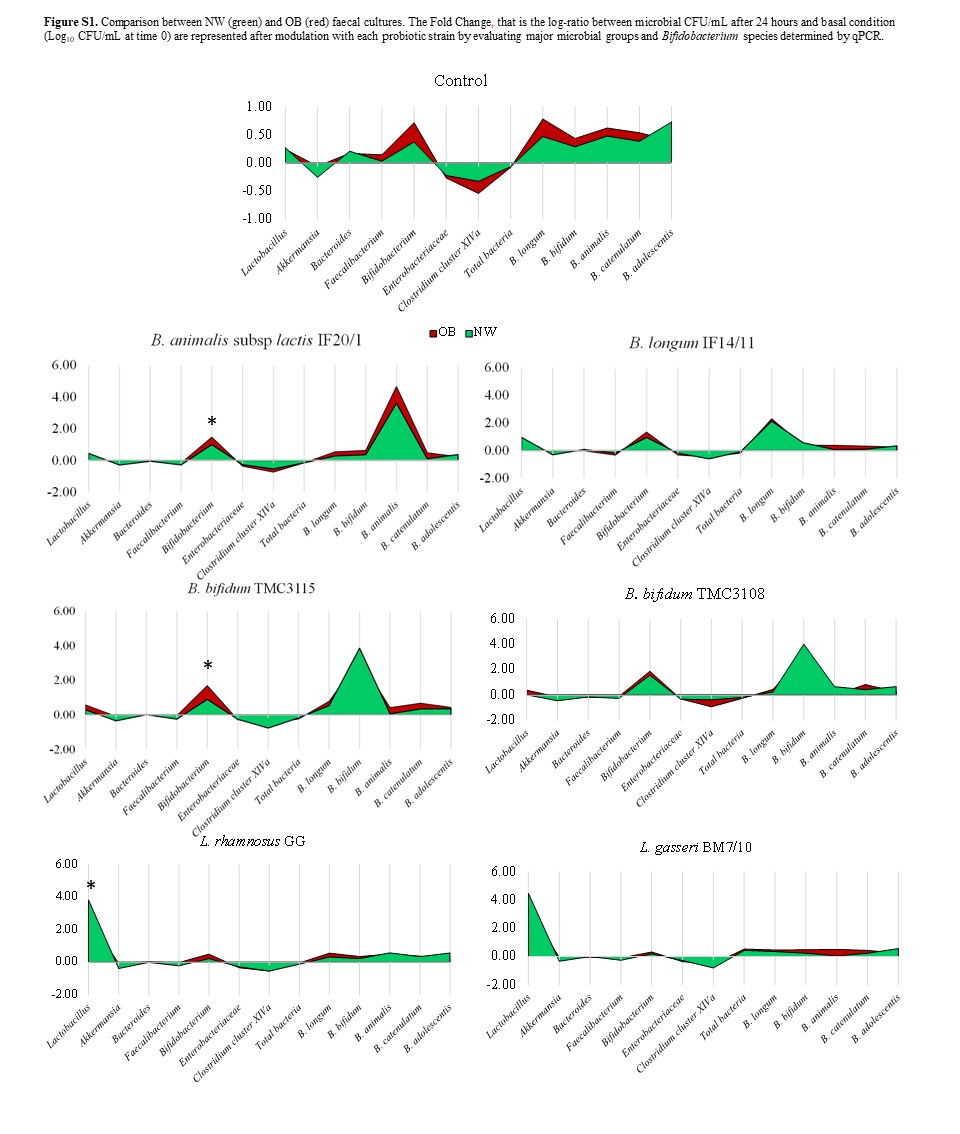

Supplement: Supplementary file 1 [file Image_1.JPEG]
