# Supplementary material for: In vitro Selection of Probiotics for Microbiota Modulation in Normal-Weight and Severely Obese Individuals: Focus on Gas Production and Interaction With Intestinal Epithelial Cells
Source: Front Microbiol. 2021 Feb 9;12:630572. doi: 10.3389/fmicb.2021.630572 (PMC7899977; doi:10.3389/fmicb.2021.630572)
Supplement: Supplementary file 2 [file Table_1.DOCX]

**Supplementary Table 1**. Absolute levels (mean ± SD) (Log_10_ CFU/mL) of major intestinal microbial groups and some *Bifidobacterium* species determined by qPCR after 24 hours of incubation with different probiotic strains.

|  | **Probiotic** | ***Clostridium* XIVa group** | **Total bacteria** | ***B. adolescentis*** | ***B. catenulatum*** | ***Akkermansia*** | ***Enterobacteria*** | ***Bacteroides* group** | ***Faecalibacterium*** |
| --- | --- | --- | --- | --- | --- | --- | --- | --- | --- |
| **OB** | **Control** | 6.70 ± 0.55 | 7.92 ^a^ ± 0.28 | 5.07 ± 1.66 | 5.67 ± 1.76 | 3.42 ± 1.04 | 7.18 ± 0.47 | 8.26 ± 0.24 | 5.69 ± 1.17 |
|  | ***B. bifidum* TMC3108** | 6.32 ± 0.09 | 7.80 ^a^ ± 0.22 | 4.90 ± 1.52 | 5.93 ± 1.36 | 3.82 ± 1.41 | 7.37 ± 0.35 | 8.00 ± 0.11 | 5.66 ± 0.44 |
|  | ***B. bifidum* TMC3115** | 6.71 ± 0.52 | 7.77 ^a^ ± 0.37 | 4.94 ± 1.59 | 5.81 ± 1.87 | 3.39 ± 0.96 | 7.16 ± 0.58 | 8.09 ± 0.25 | 5.50 ± 0.70 |
|  | ***B. animalis* IF20/1** | 6.49 ± 0.44 | 7.82 ^a^ ± 0.27 | 4.91 ± 1.61 | 5.60 ± 1.64 | 3.42 ± 1.03 | 7.07 ± 0.79 | 8.04 ± 0.27 | 5.37 ± 0.88 |
|  | ***B. longum* IF14/11** | 6.77 ± 0.38 | 7.82 ^a^ ± 0.23 | 4.93 ± 1.57 | 5.46 ± 1.59 | 3.40 ± 0.98 | 7.12 ± 0.49 | 8.06 ± 0.21 | 5.22 ± 1.31 |
|  | ***L. gasseri* BM7/10** | 6.71 ± 0.35 | 8.52 ^b^ ± 0.22 | 5.10 ± 1.70 | 5.56 ± 1.90 | 3.42 ± 1.03 | 7.09 ± 0.60 | 8.03 ± 0.24 | 5.44 ± 0.82 |
|  | ***L. rhamnosus* GG** | 6.70 ± 0.51 | 7.94 ^a^ ± 0.24 | 4.81 ± 1.60 | 5.50 ± 1.83 | 3.42 ± 1.02 | 7.11 ± 0.59 | 8.11 ± 0.30 | 5.52 ± 0.95 |
| **NW** | **Control** | 6.90 ^bc^ ± 0.26 | 8.05 ^a^ ± 0.36 | 5.62 ± 2.02 | 6.43 ± 1.97 | 4.71 ± 1.52 | 7.27 ± 0.91 | 8.31 ± 0.40 | 6.36 ± 0.67 |
|  | ***B. bifidum* TMC3108** | 7.16 ^c^ ± 0.12 | 7.97 ^a^ ± 0.18 | 5.81 ± 2.43 | 7.15 ± 1.76 | 4.72 ± 1.51 | 7.40 ± 0.12 | 8.17 ± 0.08 | 6.37 ± 0.87 |
|  | ***B. bifidum* TMC3115** | 6.46 ^a^ ± 0.48 | 7.94 ^a^ ± 0.23 | 5.24 ± 1.83 | 6.38 ± 1.94 | 4.62 ± 1.47 | 7.24 ± 0.79 | 8.16 ± 0.30 | 6.08 ± 0.68 |
|  | ***B. animalis* IF20/1** | 6.68 ^ab^ ± 0.33 | 7.95 ^a^ ± 0.31 | 5.25 ± 1.65 | 6.13 ± 1.98 | 4.66 ± 1.51 | 7.21 ± 0.76 | 8.08 ± 0.36 | 6.03 ± 0.73 |
|  | ***B. longum* IF14/11** | 6.63 ^ab^ ± 0.23 | 8.02 ^a^ ± 0.26 | 5.23 ± 1.76 | 6.14 ± 1.98 | 4.66 ± 1.47 | 7.31 ± 0.74 | 8.23 ± 0.30 | 6.14 ± 0.61 |
|  | ***L. gasseri* BM7/10** | 6.38 ^a^ ± 0.43 | 8.56 ^b^ ± 0.23 | 5.69 ± 1.72 | 6.03 ± 2.11 | 4.50 ± 1.42 | 7.20 ± 0.86 | 8.14 ± 0.23 | 6.04 ± 0.56 |
|  | ***L. rhamnosus* GG** | 6.69 ^ab^ ± 0.29 | 7.99 ^a^ ± 0.27 | 5.45 ± 1.91 | 6.38 ± 2.00 | 4.58 ± 1.45 | 7.21 ± 0.74 | 8.14 ± 0.34 | 6.10 ± 0.50 |
